# Dr. Jenner, on the Vaccine Inoculation

**Published:** 1800-06

**Authors:** Edward Jenner

**Affiliations:** New Bond Street


### *To the Editors of the Medical and Physical Journal.*

Gentlemen,

ALTHOUGH the purport of the following Answers to the Queries inserted in your last Medical and Physical Journal, has already been given in the Treatises I have published on Variolæ Vaccinæ, yet, in deference to the request of the gentleman who has proposed them, I do not hesitate in begging the favour of you to lay the following before the public; and remain,

Gentlemen,

With great respect, your obedient servant,

EDWARD JENNER.

*New Bond Street,*
*May* 15, 1800.

---

ANSWER to the first Query:

The inoculated cow-pox, taking the result of a great number of cases, appears to me, to be a disease as much milder than the inoculated small-pox, as that disease is milder than the casual small-pox.

To the 2d. I have used a variety of means to discover whether the cow-pox could be communicated by effluvia, but ineffectually.

To the 3d. A person on whom the vaccine pustule has been excited by *perfect* matter, and which has *completely* gone through the progressive stages of inflammation, maturation, and scabbing, is ever after secure from the small-pox.

To the 4th. I have seen pimples excited by the cow-pox, with sometimes a little fluid at their apex; and, in two instances, a vaccine pustule, resembling that on the arm produced by inoculation; but in no instance, a small-pox-like pustule.

To the 5th. The vaccine disease does not appear to make the least impression on the constitution unfavourable to health: on the contrary, in a great number of instances, especially among children, in whom the scrophulous diathesis has evidently been been exifting, its beneficial confequences have very foon been manifefted.

To the 6th. No peculiar difeafes have been noticed among thofe who have undergone the cow-pox at diftant periods of their lives.

The 7th and 8th. Anfwered already.

